# Associations of starchy and non-starchy vegetables with risk of metabolic syndrome: evidence from the NHANES 1999–2018

**DOI:** 10.1186/s12986-023-00760-1

**Published:** 2023-08-31

**Authors:** Yingrui Li, Bingquan Xiong, Min Zhu, Yuqian Ren, Yi Lan, Tianyang Hu, Yufan Wang, Huiping Yang, Zhiyin Liao, Kaihu Xiao, Qiang She

**Affiliations:** 1 Department of Cardiology, The Second Affiliated Hospital of Chongqing Medical University, No.74, Linjiang Road, Chonqing, 400010 China; 2 Department of Geriatrics, The First People’s Hospital of Neijiang, No. 31 Tuozhong lane, Jiaotong road, Neijiang, Sichuan 641000 China; 3 Department of Health Management Center, Chongqing University Three Gorges Hospital, No. 165 Xincheng Road, Wanzhou District, Chongqing, 404000 China; 4 Department of Pneumology, Songshan Hospital, No. 69 Star Road, Liangjiang New District, Chongqing, 401122 China; 5 Department of Precision Medicine Center, The Second Affiliated Hospital of Chongqing Medical University, No.74, Linjiang Road, Chonqing, 400010 China

**Keywords:** Metabolic syndrome, Vegetables, Potatoes, Nutrition, Risk

## Abstract

**Background:**

Higher dietary quality, including increased vegetable consumption, was associated with a reduced risk of metabolic syndrome (MetS). However, specific vegetable consumption in the development of MetS remains obscure. Our study aimed to investigate the correlation between starchy and non-starchy vegetables and MetS.

**Methods:**

Secondary data analysis from the National Health and Nutrition Examination Survey (NHANES 1999–2018). MetS was defined by National Cholesterol Education Program-Adult treatment Panel III (NCEP ATPIII) and dietary consumption was assessed by trained staff using two 24-h diet recall methods. Weighted logistic regression analysis was carried out to estimate odds ratios (ORs) and 95% confidence intervals (CIs). Subgroup analyses and restricted cubic spline (RCS) regression were performed to further investigate specific vegetable subtypes and MetS.

**Results:**

This research enrolled 24,646 individuals (11,725 females and 12,921 males), with an average age of 45.84 ± 0.23 years. Approximately 15,828(64.22%) participants were defined to be with non-MetS and 8818(35.78%) were with MetS. Both total starchy vegetables and potatoes were associated with increased MetS risk, with the corresponding OR per standard deviation (SD) (95%CI, *p*-trend) being 1.06(1.02–1.11, *p*-trend = 0.028) and 1.08(1.04–1.13, *p*-trend = 0.011), respectively. However, an inverse correlation was found between dark-green vegetables and MetS, and the OR per SD (95%CI, *p*-trend) was 0.93(0.90–0.97, *p*-trend = 0.010). Subgroup analyses showed that the positive associations of starchy vegetables and potatoes on MetS risk were stronger in non-Hispanic White participants (*p* for interaction < 0.050).

**Conclusion:**

Total starchy vegetables and white potatoes were both associated with an increased risk of MetS, while consumption of dark-green vegetables was negatively associated with MetS risk. These findings might provide a promising and healthy dietary strategy for preventing MetS.

**Supplementary Information:**

The online version contains supplementary material available at 10.1186/s12986-023-00760-1.

## Introduction

Metabolic Syndrome (MetS) is a cluster of interrelated metabolic disorders, including hypertension, dyslipidemia, glucose intolerance, and abdominal obesity, that has been linked to an increased risk of stroke, type 2 diabetes, cardiovascular disease, and other serious adverse health outcomes [1–3]. The prevalence of MetS has been increasing worldwide during the previous 20 years, with more than one billion people estimated to be affected [4, 5]. Between 1999 and 2014, the prevalence of MetS among American adults approached nearly 34%, with higher rates in non-Hispanic whites and individuals aged over 65 [5, 6]. Accordingly, MetS has emerged as a major public health concern and represents a substantial burden on human society, underscoring the need for more effective strategies to prevent and manage MetS [7].

To date, much of the research has been conducted to explore the relationships between vegetable consumption or different dietary patterns and MetS [8, 9]. However, limited data are available on the correlation between different categories of vegetables and the risk of MetS, and the existing studies have produced conflicting results [10–18]. For instance, previous studies suggested the intake of non-starchy vegetables was related to weight loss, while the intake of starchy vegetables, particularly potatoes, was linked to greater body weight [10, 11], as well as higher incidence of diabetes [12], hypertension [13], and mortality [19]. This may be due, in part, to their antioxidant loss and high glycemic index (GI) during processing [16, 20]. However, a prospective cohort study among the American adult population found that a higher intake of starchy vegetables was beneficial to health when excluding potatoes [17]. Additionally, Li et al. reported null relationships between starchy vegetable and potato intake and MetS risk [18]. Concurrently, previous studies have shown that the intake of non-starchy vegetables, especially dark-green vegetables, was inversely associated with certain cancers, depression, hepatic steatosis, and mortality [21–25]. However, the relationship with MetS has not been specifically investigated. Although current dietary guidelines generally treat all types of vegetables equally, it’s necessary to investigate potentially distinct health effects of different subgroups of vegetables [26].

Due to the existing uncertainty regarding the impact of specific vegetable consumption on MetS, we examined the correlations between starchy and non-starchy vegetable consumption as well as their subtypes and MetS risk in a nationally representative population from the US National Health and Nutrition Examination Survey (NHANES).

## Materials and methods

### Study design and population

The NHANES is a biennial and nationally replicated cross-sectional program conducted by National Center for Health Statistics (NCHS) to explore the risk factors and prevalence of common diseases among the US population. Detailed NHANES datasets were collected by highly trained staff and consisted of face-to-face interviews, biochemical tests, and physical examinations. Briefly, a complex and stratified multi-stage strategy was adopted to screen participants in NHANES [27]. All NHANES examinees were eligible to have two interviews. The first interview was completed in their home and the second was conducted in the mobile examination center (MEC) through a series of health examinations. Written informed consent was provided by all participants when they were enrolled. More detailed information about NHANES survey design, codebooks, and methods can be found at NHANES’s online website: https://www.cdc.gov/nchs/nhanes/index.htm.

**Fig. 1:**
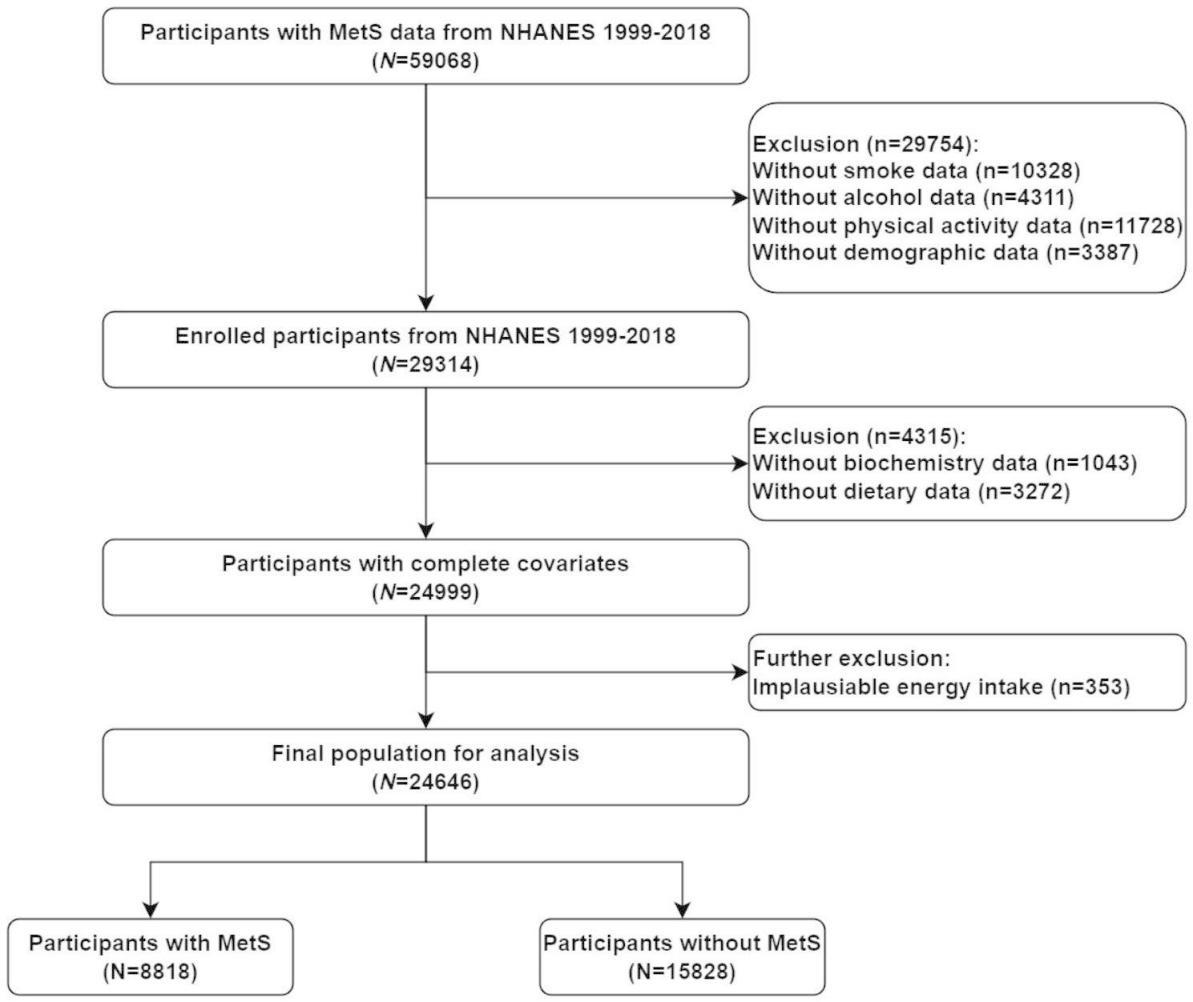
Flow diagram of the study design

In current analyses, we downloaded 10 consecutive datasets (1999–2000,2001–2002,2003–2004,2005–2006,2007–2008,2009–2010,2011–2012,2013–2014,2015–2016,2017–2018) from the NHANES website to accurately assess the relationship between different kinds of vegetables and MetS. Specifically, a total of 59,068 participants with complete MetS data were evaluated. Among them, we firstly excluded participants with the missing value of the following conditions: smoking (n = 10,328), alcohol consumption (n = 4311), physical activity (n = 11,728), and demographic data (n = 3387). Secondly, we excluded participants without biochemistry data (n = 1043) and dietary data (n = 3272). Then, we further excluded participants with unreasonable daily energy intake (≤ 500 or ≥ 5000 kcal per day, n = 353) [28]. Eventually, 24,646 eligible participants were divided into 8818 with MetS and 15,828 without MetS in the current study (Fig. 1).

### Definition of MetS

According to the previous guideline, we selected the National Cholesterol Education Program-Adult treatment Panel III (NCEP ATPIII) as the diagnostic criteria of MetS [29]. Specifically, participants were considered to be MetS if they met three or more of the following conditions: (1). Hypertriglyceridemia: serum triglyceride ≥ 150 mg/dL(1.7mmol/L), or drug treatment for elevated triglycerides; (2). Central obesity: waist circumference ≥ 102 cm in men or ≥ 88 cm in women; (3). Reduced high density cholesterols (HDL): serum HDL < 40 mg/dL(1.03mmol/L) in men and < 50 mg/dL(1.29mmol/L) in women;(4). Hypertension: systolic blood pressure (SBP) ≥ 130 mmHg or diastolic blood pressure (DBP) ≥ 85 mmHg or drug treatment for hypertension;(5). Hyperglycemia: fasting glucose ≥ 100 mg/dL(5.6mmol/L) or drug treatment for elevated blood glucose. For blood pressure measurement, all participants were allowed to rest quietly for five minutes in MEC and determine the maximum inflation level. In the current study, we obtained three consecutive measurement of blood pressure, and then calculated the arithmetic mean of blood pressure to obtain the final recording.

### Dietary assessment

In the current study, data on dietary intake were obtained using two 24-hour dietary recall surveys. All NHANES examinees were eligible to take part in these interviews. The first 24-hour recall survey was conducted face-to-face by trained food recall data collectors in the interview room of MEC, then the second was carried out over the phone 3–10 days later. In our statistical analysis, dietary intake data were completed for all participants. Therefore, the average vegetable intake over 2 days was used in this statistical analysis to reduce bias.

Based on the 2017–2018 American Food Pattern Equivalent Database (FPED), we defined different types of vegetables (Additional file 1: Table S1). White potatoes (such as fried, boiled, baked potatoes and potatoes and potato chips) and other starchy vegetables (such as unripe peas, lima beans, corn, breadfruit, burdock, and cassava) are all starchy vegetables. Dark-green vegetables (such as arugula, basil, beet greens, bitter melon leaves, and broccoli), red and orange vegetables (such as carrots, red peppers, red or orange bell peppers, and squash), and other non-starchy vegetables (such as artichokes, asparagus, avocado, bamboo shoots, and beets) are all non-starchy vegetables.

### Covariates

Age, gender, race, marital status, poverty income ratio (PIR), education, smoking, alcohol status, physical activity, serum creatinine, uric acid, alanine aminotransferase (ALT), aspartate aminotransferase (AST), and intake of energy, carbohydrate, whole grains, meat, and nuts were considered as covariates that associated with MetS. Age was treated as continuous variable. Race was stratified into Non-Hispanic Black, Non-Hispanic White, Mexican American, and other races. PIR was stratified into three levels: <1.30, 1.30–3.49, and ≥ 3.50. Marital status was grouped into married and unmarried. Education was stratified into 5 levels: less than 9th grade, 9th-11th grade, high school, some college, and college or above. Based on prior literature’s definition of alcohol consumption, we divided participants into four groups:1) Never drinking: no history of alcohol consumption or former drinkers, 2) Current heavy drinking (≥ 3 drinks/day for women, ≥ 4 drinks/day for men),3) current moderate drinking (≥ 2 drinks/day for women, ≥ 3 drinks/day for men,4) current light drinking: does not meet above [30]. In addition, physical activity was calculated as metabolic equivalent (MET)-minutes/week and classified into 3 categories:<600, 600–1200, and ≥ 1200 MET- minute/week [31].

### Statistical analysis

According to NHANES analysis tutorial, a complex survey design was fully considered and dietary 2-day sample weight was applied in the current analyses. Continuous variables were presented as mean and standard error (SE), and categorical variables were expressed as the frequency with percentage. Chi-square test and Student’s *t*-test were carried out to assess individuals’ baseline characteristics according to MetS status. Based on previously published literature [14], the standard intake of all types of vegetables was 80 g, thus vegetable intake in the current study was stratified into three groups:0 serving/day, < 2 servings/day, and ≥ 2 servings/day. We established three multivariable logistic regression models to analyze the risk of MetS and different kinds of vegetables. Model 1 was a crude model for none variables adjustment. Model 2 was adjusted for age, gender, and race. Model 3 was further adjusted for model 2, plus marital status, PIR, education, smoking, alcohol status, physical activity, serum creatinine, uric acid, ALT, AST, and intake of energy, carbohydrate, whole grains, meat, and nuts. Additionally, we also calculate the odds ratios (ORs) for per standard deviation (SD) increment of different vegetables in these models. We further tested the linear trend by using each vegetable group as an ordinal variable. The potential nonlinear relationship between vegetables and MetS risk was investigated by restricted cubic spline (RCS) analysis. In the RCS model, we adjusted for all the confounding factors mentioned above. Subgroup analyses were used to investigate the stratified correlation between vegetables and MetS risk in different populations. Finally, the likelihood ratio test was used to check the interaction among these subgroups.

Furthermore, we examined the association of specific vegetables with MetS components in a fully-adjusted model to further identify the influence of vegetables on lipid metabolism, glucose metabolism, and blood pressure. All participants with missing values were excluded from the current analysis. The significance level was defined as α = 0.05 and all statistical tests were bilateral. R software (version 4.1.3) was conducted for all statistical analyses.

## Results

### Characteristics of participants

**Fig. 2:**
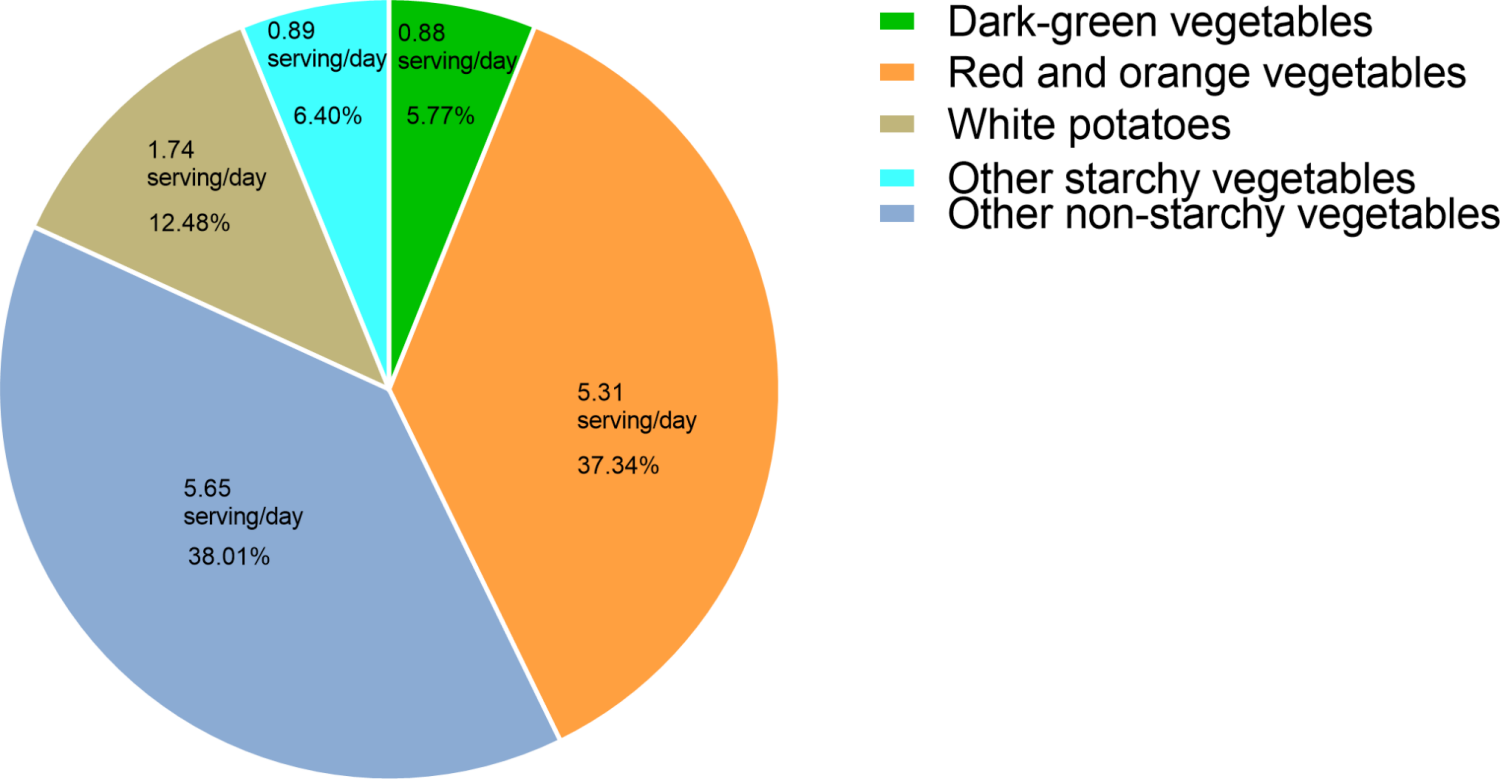
Proportions of specific vegetable intake that contributes to total vegetable intake

A total of 24,646 eligible participants were enrolled in our study, representing approximately 137.57 million US population. Detailed baseline characteristics are shown in Table 1. Specifically, variables of age, gender, race, education, PIR, marital status, serum creatinine, smoke, and physical activity were all associated with intake of total starchy vegetables and non-starchy vegetables (all *p* < 0.050). Of the 24,646 study participants, 11,725(47.57%) were females and 12,921(52.43%) were males, with a mean age of 45.84 ± 0.23 years old. Approximately 15,828(64.22%) participants were defined to be with non-MetS and 8818(35.78%) were with MetS. Compared with the non-MetS group, participants with MetS were more likely to be older, male, Non-Hispanic White, non-college-educated, married, have a lower level of PIR, and have a higher level of serum creatinine, uric acid, ALT, and AST. In addition, participants with MetS tend to be inactive in physical activity, be non-smokers and non-drinkers, have higher meat and carbohydrate intake, and have lower intake of fruits, energy, whole grains, and nuts (all *p* < 0.050). There was no significant difference between the two groups in consumption of fat, refined grains, and coffee (all *p* > 0.050, Additional file 1: Table S2). Intake of red and orange vegetables and other non-starchy vegetables accounted for a significant portion of total vegetable intake, with 5.31 servings/day (37.34%) of the intake coming from red and orange vegetables, 5.65 servings/day (38.01%) from other non-starchy vegetables, 1.74 servings/day (12.48%) from white potatoes, 0.89serving/day (6.40%) from other starchy vegetables and 0.88 serving/day (5.77%) from dark green vegetables (Fig. 2).

**Table 1:** Characteristics of study participants according to individual vegetable intake from NHANES 1999–2018, weighted(n = 24,646)^a^

	Total starchy vegetables(servings/day)				Total non-starchy vegetables(servings/day)			
variables	0	< 2	≥ 2	*P*	0	< 2	≥ 2	*P*
Age, years	44.91 ± 0.31	46.07 ± 0.34	46.50 ± 0.30	< 0.001	44.15 ± 0.63	46.28 ± 0.60	45.88 ± 0.24	0.024
BMI, kg/m^2^	28.26 ± 0.11	28.34 ± 0.14	28.60 ± 0.13	0.076	28.85 ± 0.30	28.67 ± 0.22	28.38 ± 0.09	0.146
Gender, n (%)				< 0.001				0.016
Female	3978(49.54)	3378(53.80)	4369(44.37)		455(46.62)	951(52.87)	10,319(48.39)	
Male	4351(50.46)	3046(46.20)	5524(55.63)		609(53.38)	905(47.13)	11,407(51.61)	
Race, n (%)				< 0.001				< 0.001
Mexican American	1412(7.75)	879(6.21)	1523(7.31)		103(4.93)	247(4.79)	3464(7.44)	
Non-Hispanic Black	1484(8.60)	1336(9.96)	1742(8.91)		306(15.77)	459(13.91)	3797(8.44)	
Non-Hispanic White	4190(73.51)	3353(74.18)	4905(71.77)		516(67.60)	933(72.97)	10,999(73.23)	
Other races	1243(10.14)	856(9.64)	1723(12.01)		139(11.70)	217(8.33)	3466(10.89)	
Education, n (%)				0.022				< 0.001
Less than 9th grade	763(3.93)	405(2.89)	747(3.62)		104(5.26)	194(5.39)	1617(3.32)	
9th-11th grade	1009(8.62)	745(8.12)	1213(9.16)		202(16.38)	267(11.98)	2498(8.13)	
High school	1797(21.83)	1510(23.27)	2334(23.18)		297(30.86)	508(26.40)	4836(22.12)	
Some college	2554(32.44)	2017(32.04)	3067(33.08)		328(32.62)	553(33.99)	6757(32.48)	
College or above	2206(33.18)	1747(33.67)	2532(30.97)		133(14.88)	334(22.24)	6018(33.95)	
PIR, n (%)				< 0.001				< 0.001
< 1.30	2298(19.82)	1534(16.50)	2620(17.97)		409(33.96)	547(23.09)	5496(17.18)	
1.30–3.49	3039(32.15)	2360(33.39)	3822(36.24)		386(35.49)	742(36.92)	8093(33.82)	
≥ 3.50	2992(48.03)	2530(50.11)	3451(45.78)		269(30.56)	567(39.99)	8137(49.00)	
Marital status, n (%)				< 0.001				< 0.001
Unmarried	3967(45.22)	2785(40.08)	4294(41.54)		606(56.86)	889(45.45)	9551(41.56)	
Married	4362(54.78)	3639(59.92)	5599(58.46)		458(43.14)	967(54.55)	12,175(58.44)	
Serum creatinine, umol/L	77.43 ± 0.39	77.05 ± 0.35	79.27 ± 0.31	< 0.001	82.82 ± 2.02	76.89 ± 0.68	77.94 ± 0.23	< 0.001
Uric acid, µmol/L	321.55 ± 1.34	318.41 ± 1.36	326.43 ± 1.41	< 0.001	329.55 ± 3.99	320.94 ± 2.72	322.48 ± 0.98	0.197
ALT, U/L	25.89 ± 0.35	25.34 ± 0.35	26.32 ± 0.30	0.115	25.19 ± 0.80	26.76 ± 1.40	25.88 ± 0.17	0.559
AST, U/L	25.58 ± 0.26	25.14 ± 0.28	25.44 ± 0.22	0.513	25.10 ± 0.53	25.19 ± 0.45	25.44 ± 0.14	0.726
Smoke, n (%)	1837(21.61)	1255(19.21)	1922(19.38)	0.023	370(36.19)	472(25.91)	4172(18.97)	< 0.001
Physical activity (MET- minute/week), n (%)				0.001				< 0.001
< 600	2918(33.16)	2450(37.25)	3378(33.28)		392(35.84)	850(45.78)	7504(33.36)	
600–1200	1236(14.33)	945(14.73)	1513(14.62)		149(14.72)	268(13.19)	3277(14.65)	
≥ 1200	4175(52.51)	3029(48.01)	5002(52.09)		523(49.44)	738(41.02)	10,945(51.99)	
Alcohol status, n (%)				0.057				< 0.001
Never	2189(21.05)	1735(21.83)	2783(23.52)		358(29.09)	610(27.10)	5739(21.58)	
Mild	3007(37.55)	2334(38.75)	3549(37.22)		304(27.64)	630(35.42)	7956(38.34)	
Moderate	1369(18.74)	1132(18.86)	1555(17.65)		136(13.86)	276(17.14)	3644(18.62)	
Heavy	1764(22.66)	1223(20.56)	2006(21.61)		266(29.41)	340(20.34)	4387(21.46)	

Continuous variables were shown as mean ± SE, categorical variables were shown as frequency(percentage)

^a^All estimates accounted for complex survey designs, and all percentages were weighted

Abbreviations: MetS, metabolic syndrome; PIR, poverty income ratio; ALT, alanine aminotransferase; AST, aspartate aminotransferase; MET, metabolic equivalent; SE, standard error

### Association between vegetables and MetS

**Fig. 3:**
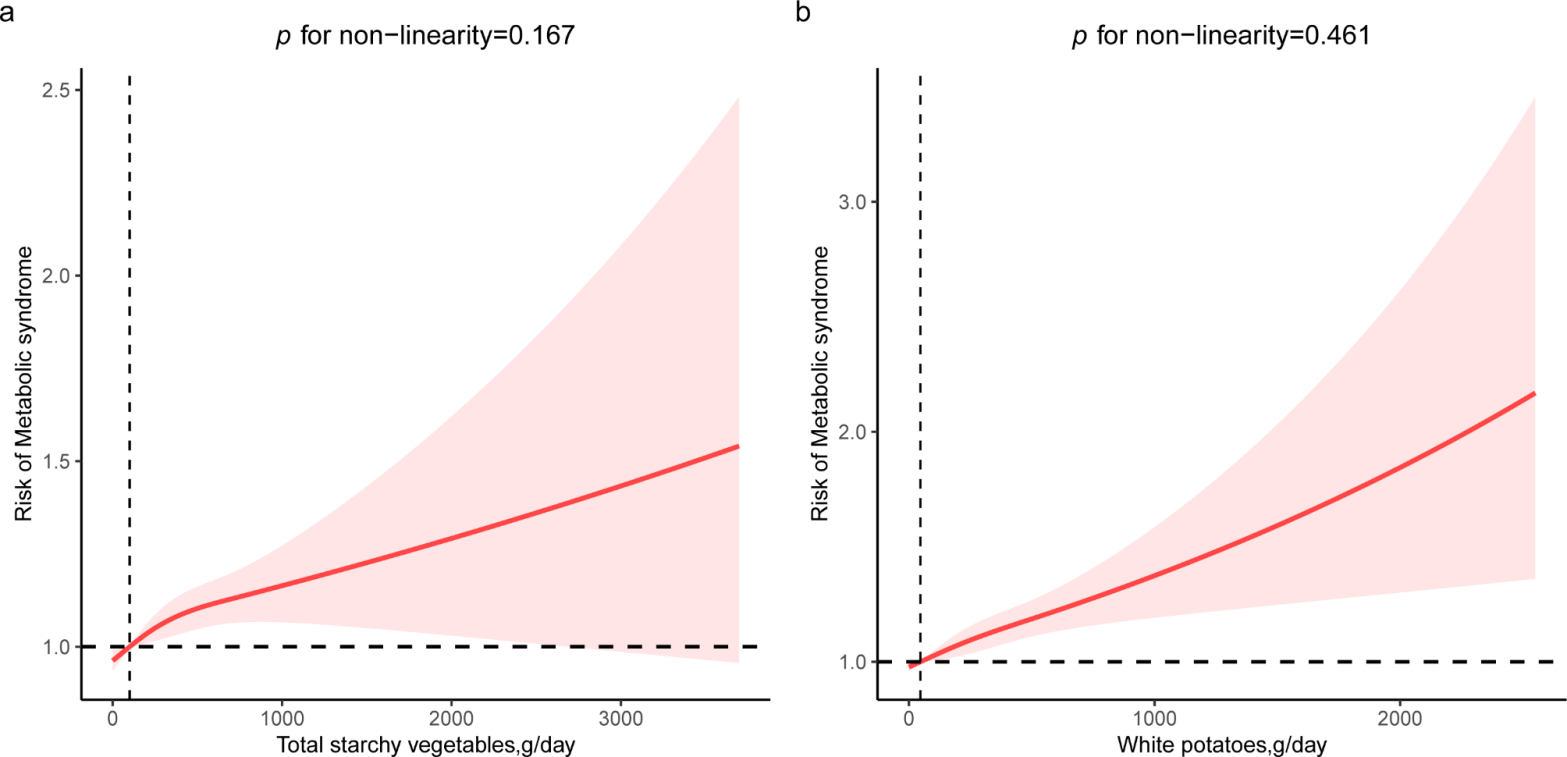
Restricted cubic spline analysis (RCS) with multivariate-adjusted associations between starchy vegetables and the risk of MetS. (**a**) RCS analysis between total starchy vegetable intake and MetS risk. (**b**) RCS analysis between white potatoes intake and MetS risk Age, gender, race, marital status, PIR, BMI, education, smoking, alcohol status, physical activity, serum creatinine, uric acid, ALT, AST, energy, fruits, carbohydrate, whole grains, meat, nuts, and non-starchy vegetables. Of note, other starchy vegetables were further adjusted in RCS analysis for white potatoes

After adjusting for all confounders, consuming two or more servings of daily total starchy vegetables was related to a higher risk of MetS compared with consuming none (OR = 1.14,95%CI: 1.02–1.28, *p* for trend = 0.028), and the ORs(95%CI) per SD increment was 1.06(1.02–1.11) (Table 2). Furthermore, results displayed that higher white potato intake was linked to a higher risk of MetS (OR = 1.16,95%CI:1.04–1.30, *p* for trend = 0.011) with the corresponding ORs(95%CI) per SD increment was 1.08(1.04–1.13) (Table 2). However, no association between other starchy vegetable intake and MetS risk was found. In the multivariate-adjusted RCS model, we further observed a linear positive association between total starchy vegetables and MetS risk, and the non-linear *p* was 0.167 (Fig. 3). A similar positive relationship between white potatoes and the risk of MetS was observed for the same comparison. Consuming ≥ 2 servings/day of dark-green vegetables was associated with reduced risk of MetS when compared with participants consuming none (OR = 0.86, 95%CI:0.76–0.98, *p* for trend = 0.010), and the corresponding OR (95%CI) per SD increment was 0.93 (0.90,0.97) (Table 3). No significant associations of total non-starchy vegetables, red and orange vegetables, and other non-starchy vegetables on MetS were observed in the fully-adjusted model.

**Table 2:** Association between starchy vegetable consumption and MetS risk

	Category of starchy vegetable intake				*P* trend
	0 serving/day	< 2 servings/day	≥ 2 servings/day	Per SD increase	
Total starchy vegetables (0-6249.13 g/day)					
No. of participants	8329	6424	9893		
Model 1	Ref	1.03(0.92,1.14)	1.23(1.11,1.35)	1.12(1.07,1.16)	< 0.001
Model 2	Ref	1.00(0.90,1.12)	1.16(1.05,1.28)	1.09(1.05,1.14)	0.004
Model 3	Ref	0.98(0.88,1.10)	1.14(1.02, 1.28)	1.06(1.02,1.11)	0.028
**White potatoes** **(0-3828.25 g/day)**					
No. of participants	10,745	6430	7471		
Model 1	Ref	1.08(0.98,1.20)	1.28(1.17,1.41)	1.12(1.08,1.17)	< 0.001
Model 2	Ref	1.08(0.97,1.20)	1.23(1.12,1.36)	1.12(1.07,1.16)	< 0.001
Model 3	Ref	1.04(0.93,1.16)	1.16(1.04,1.30)	1.08(1.04,1.13)	0.011
**Other starchy vegetables** **(0-3568.88 g/day)**					
No. of participants	17,646	3067	3933		
Model 1	Ref	1.09(0.97,1.22)	1.12(1.01,1.24)	1.05(1.01,1.09)	0.020
Model 2	Ref	0.97(0.86,1.10)	1.01(0.90,1.12)	1.02(0.98,1.06)	0.921
Model 3	Ref	0.97(0.85,1.10)	0.98(0.87,1.10)	0.99(0.95,1.04)	0.615

Model 1: None

Model 2: Age, gender, race

Model 3: Age, gender, race, marital status, PIR, BMI, education, smoking, alcohol status, physical activity, serum creatinine, uric acid, ALT, AST, energy, fruits, carbohydrate, whole grains, meat, nuts, and non-starchy vegetables. Of note, potatoes and other starchy vegetables were mutually adjusted

**Table 3:** Association between non-starchy vegetable consumption and MetS risk

	Category of non-starchy vegetable intake				*P* trend
	0 serving/day	< 2 servings/day	≥ 2 servings/day	Per SD increase	
Total non-starchy vegetables (0-10791.56 g/day)					
No. of participants	1064	1856	21,726		
Model 1	Ref	0.95(0.76,1.20)	0.88(0.75,1.04)	1.01(0.97,1.05)	0.039
Model 2	Ref	0.88(0.70,1.12)	0.81(0.68,0.95)	1.03(0.98,1.07)	0.003
Model 3	Ref	0.92(0.72,1.18)	0.89(0.74,1.07)	1.03(0.98,1.08)	0.175
**Dark-green vegetables** **(0-3819.19 g/day)**					
No. of participants	16,707	4023	3916		
Model 1	Ref	0.85(0.77,0.94)	0.77(0.69,0.86)	0.93(0.89,0.97)	< 0.001
Model 2	Ref	0.77(0.69,0.86)	0.73(0.65,0.81)	0.92(0.88,0.97)	< 0.001
Model 3	Ref	0.89(0.79,1.00)	0.86(0.76,0.98)	0.93(0.90,0.97)	0.010
**Red and orange vegetables** **(0-7105.43 g/day)**					
No. of participants	3127	4922	16,597		
Model 1	Ref	0.89(0.78,1.02)	0.89(0.80,1.00)	1.03(0.98,1.08)	0.110
Model 2	Ref	0.90(0.78,1.04)	0.91(0.80,1.03)	1.05(1.00,1.10)	0.231
Model 3	Ref	0.98(0.83,1.16)	1.01(0.87, 1.17)	1.05(0.99,1.11)	0.088
**Other non-starchy vegetables** **(0-8470.33 g/day)**					
No. of participants	2401	3942	18,303		
Model 1	Ref	1.00(0.85,1.17)	0.97(0.85,1.10)	1.02(0.98,1.06)	0.485
Model 2	Ref	0.91(0.77,1.08)	0.87(0.76,0.99)	1.03(0.99,1.07)	0.028
Model 3	Ref	0.94(0.78,1.13)	0.93(0.80,1.08)	1.04(0.99,1.10)	0.409

Model 1: None

Model 2: Age gender, race

Model 3: Age, gender, race, marital status, PIR, BMI, education, smoking, alcohol status, physical activity, serum creatinine, uric acid, ALT, AST, energy, fruits, carbohydrate, whole grains, meat, nuts, and starchy vegetables. Of note, dark-green vegetables, red and orange vegetables, and other non-starchy vegetables were mutually adjusted

### Relation between individual MetS components and specific vegetables

Table 4 summarized the results of specific vegetables on an individual component of MetS after controlling all covariates. Participants consuming ≥ 2 servings/day total starchy vegetables were 1.13-fold odds of hyperglycemia (OR = 1.13,95%CI:1.01,1.26) than participants consuming none total starchy vegetables. Similarly, intake of white potatoes ≥ 2 servings/day was related to increased risk of hyperglycemia and central obesity, with the corresponding ORs(95%CIs) being 1.15(1.03,1.28) and 1.22(1.08,1.38), respectively. However, dark-green vegetables were related to decreased risk of reduced HDL(OR = 0.83,95%CI:0.76,0.92), central obesity (OR = 0.77,95%CI:0.67,0.89), and hypertension (OR = 0.87,95%CI:0.76,1.00).

**Table 4:** Association between specific vegetable consumption and individual MetS component

	0 serving/day	< 2 servings/day		≥ 2 servings/day	
Total starchy vegetables^a^ (0-10791.56 g/day)		OR (95%CI)	*P*	OR (95%CI)	*P*
Hyperglycemia	Ref	1.01(0.88,1.15)	0.917	1.13(1.01,1.26)	0.033
Reduced HDL	Ref	1.05(0.94,1.17)	0.407	1.10(0.99,1.23)	0.082
Hypertriglyceridemia	Ref	0.94(0.84,1.06)	0.328	1.05(0.95,1.15)	0.363
Central obesity	Ref	0.99(0.87,1.13)	0.916	1.04(0.93,1.17)	0.502
Hypertension	Ref	1.05(0.94,1.18)	0.392	1.08(0.97,1.21)	0.151
**White potatoes** ^**b**^ **(0-3828.25 g/day)**					
Hyperglycemia	Ref	1.06(0.92,1.21)	0.414	1.15(1.03,1.28)	0.015
Reduced HDL	Ref	1.02(0.92,1.12)	0.727	1.11(0.99,1.23)	0.062
Hypertriglyceridemia	Ref	0.98(0.87,1.09)	0.669	1.08(0.97,1.19)	0.144
Central obesity	Ref	1.08(0.94,1.23)	0.278	1.22(1.08,1.38)	0.002
Hypertension	Ref	1.08(0.97,1.20)	0.176	1.11(0.99,1.24)	0.063
**Dark-green vegetables** ^**c**^ **(0-3819.19 g/day)**					
Hyperglycemia	Ref	0.96(0.84,1.09)	0.490	0.98(0.88,1.10)	0.759
Reduced HDL	Ref	0.87(0.77,0.99)	0.032	0.83(0.76,0.92)	< 0.001
Hypertriglyceridemia	Ref	0.94(0.84,1.06)	0.301	0.90(0.80,1.01)	0.081
Central obesity	Ref	0.87(0.76,1.01)	0.070	0.77(0.67,0.89)	< 0.001
Hypertension	Ref	0.90(0.79,1.02)	0.108	0.87(0.76,1.00)	0.044

^a^Adjusted for age, gender, race, marital status, PIR, BMI, education, smoking, alcohol status, physical activity, serum creatinine, uric acid, ALT, AST, energy, fruits, carbohydrate, whole grains, meat, nuts, and non-starchy vegetables

^b^Adjusted for age, gender, race, marital status, PIR, BMI, education, smoking, alcohol status, physical activity, serum creatinine, uric acid, ALT, AST, energy, fruits, carbohydrate, whole grains, meat, nuts, non-starchy vegetables, and other starchy vegetables

^c^Adjusted for age, gender, race, marital status, PIR, BMI, education, smoking, alcohol status, physical activity, serum creatinine, uric acid, ALT, AST, energy, fruits, carbohydrate, whole grains, meat, nuts, starchy vegetables, red and orange vegetables, and other non-starchy vegetables

### Subgroup analyses

**Fig. 4:**
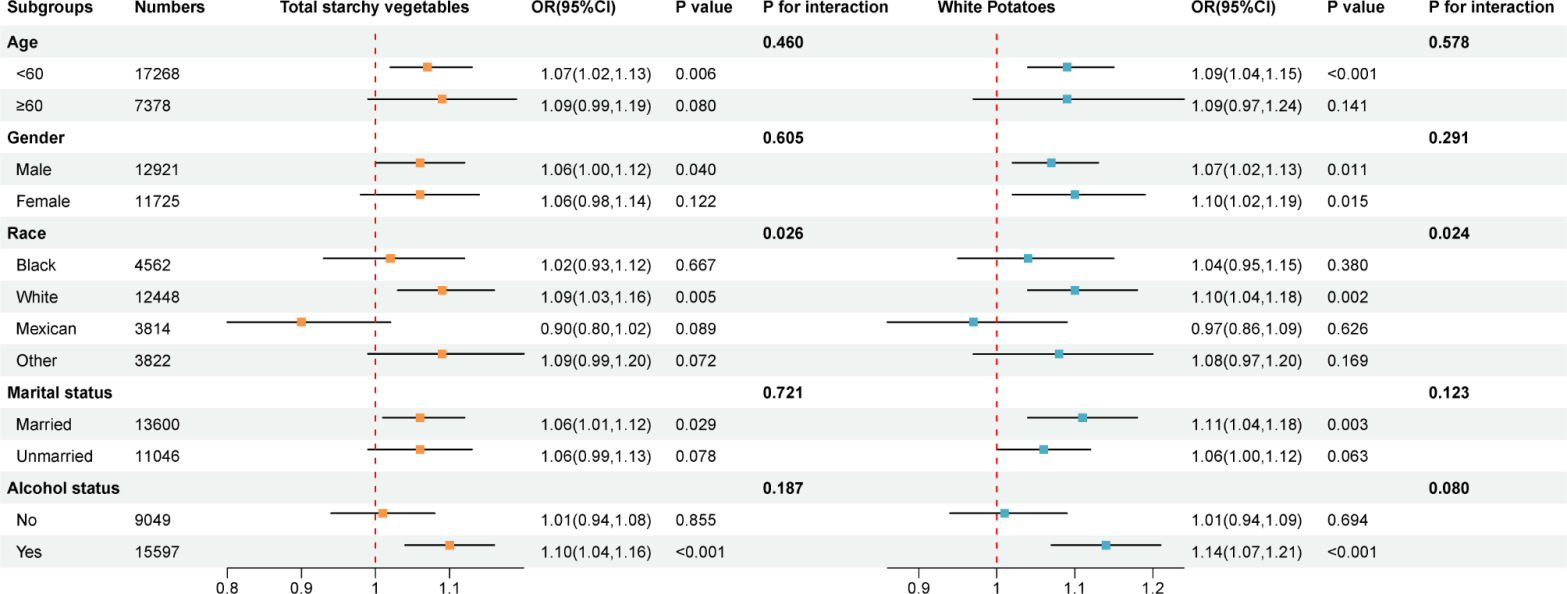
Subgroup analysis of the association between starchy vegetables (per SD increase) and MetS risk Age, gender, race, marital status, PIR, BMI, education, smoking, alcohol status, physical activity, serum creatinine, uric acid, ALT, AST, energy, fruits, carbohydrate, whole grains, meat, nuts, and non-starchy vegetables. Of note, other starchy vegetables were further adjusted in subgroup analysis for white potatoes

Subgroup analyses were performed to further understand the association between vegetables and MetS risk among different populations. As shown in Fig. 4, we observed both total starchy vegetables and white potatoes were positively associated with MetS risk in non-Hispanic White participants, and a significant interaction was found (both *p* for interaction < 0.050).

## Discussion

In this large observational research among American adults, we found consumption of total starchy vegetables and white potatoes displayed a positive correlation with an increased risk of MetS, whereas consumption of dark green vegetables demonstrated a negative correlation with a decreased risk of MetS. Subgroup analyses revealed that these affirmative associations were even more pronounced in the non-Hispanic white population. Although current dietary guidelines generally treat all types of vegetables equally, these findings highlight potentially distinct health effects of different subgroups of vegetables [32].

It is noteworthy that most previous studies primarily focused on dietary patterns or total vegetable consumption without considering the potential impact that particular vegetable subtypes on MetS. A study conducted in Tehran revealed that sufficient vegetable consumption could potentially lower the incidence of MetS in children and adolescents [33]. A meta-analysis of 26 observational studies reported that vegetable intake was negatively related to MetS [8], whereas another meta-analysis of 8 randomized controlled trials suggested that vegetable consumption was only associated with the decrease of diastolic blood pressure, but did not affect other MetS components. Until now, the relationship between intake of starchy vegetables and MetS remains controversial. A cross-sectional study conducted in Costa Rica found that consuming more starchy vegetables may not be related to the increasing prevalence of MetS [18], but a cohort study from China showed a positive association between total carbohydrate intake from starchy foods and MetS [34], which was in line with our findings. This discrepancy could be explained in part by differences in dietary patterns, as well as the exclusion of unhealthy starchy vegetables in the Costa Rica study, which were defined as potatoes including baked/boiled/mashed potatoes, French fries, and potato chips. Costa Rica’s dietary patterns emphasize fresh vegetables, legumes, and cereals as the main sources of starchy vegetable intake, with less red meat consumption [35, 36]. In contrast, the Western diet in the United States includes less favorable choices such as French fries, potato chips, and mashed potatoes, often accompanied by increased intake of processed meat, red meat, and refined flour [37]. Further multicenter prospective cohort studies are required to better understand the association between different types of vegetables and MetS.

Potatoes were widely consumed starchy food in many countries, as well as a good source of fiber and vitamins like folate, potassium, iron, vitamin B6, and vitamin C [38]. Remarkably, in contrast to the US Dietary Guidelines, the World Health Organization, and some previous studies did not consider potatoes to be vegetables [39, 40]. White potato intake, especially fried potatoes, has been linked to weight gain, hypertension, type 2 diabetes, and mortality in previous research [13, 41–43]. On the contrary, another study in 1881 Costa Rican adults found no conclusive links between increased potato consumption and the incidence of MetS [18]. However, in our study, white potatoes were positively associated with MetS. As part of an unhealthy Western dietary pattern, white potatoes were commonly served as French fries, potato chips, and mashed potatoes, which could help to explain the positive association between white potatoes and MetS.

According to our findings, there was a positive significant association between total starchy vegetables and hyperglycemia. A similar positive association of white potatoes with hyperglycemia and central obesity was found. Some biological mechanisms might be able to explain our findings. Numerous pieces of evidence have demonstrated that insulin resistance exerts a pivotal role in the pathogenesis of MetS [44]. Starchy vegetables are generally able to quickly elevate blood glucose levels, thereby increasing glycemic load and insulin response [23]. The glycemic index was altered by the variety of potato, as well as how it was cooked or processed [45]. The average GI value of tested potatoes in North America was 67 ± 16, which was lower than those of Australia and Europe. Phy et al. observed that low-starch diets provided benefits such as weight loss, lower blood lipid levels, and enhanced insulin sensitivity [46]. By contrast, non-starchy vegetables contain dietary fiber that can aid in lowering blood sugar, cholesterol, and weight loss [47].

Furthermore, the occurrence and progression of metabolic syndrome are partly influenced by oxidative stress. Several antioxidant nutrients, flavonoids, minerals, dietary fiber, phytochemicals, and phenols found in non-starchy vegetables, particularly green leafy vegetables, can lessen deoxyribonucleic acid (DNA) damage and oxidative stress brought on by free radicals [48], as well as vivo lipid peroxidation [49]. Folic acid, magnesium, and vitamin B2 acid are all abundant in dark-green vegetables. In a prospective cohort from the CARDIA study, sufficient intake of folic acid, vitamin B6, and vitamin B12 might help prevent metabolic syndrome [50]. Magnesium has been demonstrated to have antioxidant, anti-inflammatory, and anti-diabetic capabilities [51], and it was found to be significantly inversely related to the occurrence of MetS [52, 53]. Potatoes are also rich in antioxidant ingredients, such as chlorogenic acids and ascorbic [54]. Simultaneously, the antioxidative potency during the peeling and cooking phases may experience a marked diminution [16]. According to reports, the amount of antioxidants in potatoes varies significantly depending on genotype, environment, and planting year. In comparison to pigmented potatoes such as yellow and purple potatoes, the consumption of white potatoes may not reduce DNA oxidative damage and inflammation [55].

The development of MetS is also accompanied with chronic low-grade inflammation [9, 56]. Kerrie L et al. enrolled healthy male adult participants from Washington and found that consumption of white potatoes rather than yellow or purple potatoes could increase C-reactive protein (CRP) and Interleukin 6 (IL-6) concentrations [55]. Apart from potatoes, a higher quantity of vegetables and fruits was linked to decreased CRP plasma concentrations [39]. A proinflammatory nutritional pattern, which included potatoes, was created by Janett et al. and was linked to circulating levels of CRP and IL-6 [57]. Additionally, the consumption of potato chips may elevate CRP and IL-6 levels, probably due to acrylamide, which is a vital cellular antioxidant and can decrease glutathione stores [58]. However, the Mediterranean diet, which is abundant in fruit and vegetables, has been reported to reduce plasma markers of inflammation and endothelial dysfunction [59].

The observed positive association between total starchy vegetables and white potatoes with MetS risk in non-Hispanic White participants could be due to several factors, including lifestyle, dietary patterns, and genetic predisposition. Non-Hispanic whites were reported to have dietary habits and preferences characterized by lower fruit and vegetable consumption, along with a higher intake of potatoes, bread, and vegetables paired with butter, margarine, or fats [60]. From 2011 to 2018, non-Hispanic whites showed a significant decrease in their HEI-2015 score [61], indicating diet quality trends varied across different race/ethnicity groups. Alternatively, the outcomes might be the consequence of chance. Additional research is warranted to confirm these findings and clarify the underlying mechanisms.

Our study has several noteworthy advantages. First of all, our study data is established on the NHANES database, which provides a large-scale and nationally representative database, making our study findings reliable. Second, our study is the first study to explore the potential impact of starchy vegetables and non-starchy vegetables on MetS, which has not been investigated in other studies. Third, analyses investigating the association of specific vegetables with individual MetS components are performed to provide robustness to our results.

Of note, several limitations should be pointed out in our research. In fact, the nature of the cross-sectional study precludes us from establishing causality between the vegetables and MetS, thus future longitudinal studies are needed. In addition, although our analysis provides details on vegetable intake, we regrettably omitted the consideration of various cooking methods in our analysis. Prior studies have indicated that disparate cooking or processing techniques might exert an impact on the nutrient composition and glycemic index (GI) of potatoes [62]. Moreover, the dietary intake survey was collected through 24-h recall interviews, which might have recall bias and not accurately reflect the daily intake of U.S. adults. Finally, although some conventional variables are fully controlled in the current study, other unmeasured confounders are not considered.

## Conclusion

In summary, the current study findings revealed that the consumption of total starchy vegetables and white potatoes were both positively correlated with an increased risk of MetS, whereas the intake of dark-green vegetables exhibited a negative association with the risk of MetS. These findings provide a promising and healthy strategy for public to prevent MetS. Subsequent prospective cohort investigations are imperative to ascertain the causality of these findings and to delve into the potential underlying mechanisms.

### Electronic supplementary material

Below is the link to the electronic supplementary material.

**Additional file 1**. **Table S1**. The definitions of starchy and non-starchy vegetables according to FPED 2017-2018. **Table S2**. Characteristics of study participants by MetS status, weighted (n=24646)^a^

## Data Availability

The datasets generated and/or analyzed are available from the corresponding. author on reasonable request.

## Abbreviations

ALT: Alanine aminotransferase
AST: Aspartate aminotransferase
CI: Confidence interval
CRP: C-reactive protein
DBP: Diastolic blood pressure
DNA: Deoxyribonucleic acid
FPED: Food Pattern Equivalent Database
GI: Glycemic index
HDL: High density cholesterols
IL-6: Interleukin 6
MEC: Mobile examination center
MetS: Metabolic syndrome
NCEP ATPIII: National Cholesterol Education Program-Adult treatment Panel III
NCHS: National Center for Health Statistics
NHANES: National Health and Nutrition Examination Survey
OR: Odds ratio
PIR: Poverty income ratio
RCS: Restricted cubic spline
SBP: Systolic blood pressure
SD: Standard deviation
SE: Standard error
US: United States
