# Supplementary material for: Associations of starchy and non-starchy vegetables with risk of metabolic syndrome: evidence from the NHANES 1999–2018
Source: Nutr Metab (Lond). 2023 Aug 31;20:36. doi: 10.1186/s12986-023-00760-1 (PMC10472713; doi:10.1186/s12986-023-00760-1)
Supplement: Supplementary file 1 — Additional file 1. Table S1. The definitions of starchy and non-starchy vegetables according to FPED 2017-2018. Table S2. Characteristics of study participants by MetS status, weighted (n=24646)a [file 12986_2023_760_MOESM1_ESM.docx]

**Tables**

**Table S1.** The definitions of starchy and non-starchy vegetables according to FPED 2017-2018

| **Foods or food groups** | **Food items** |
| --- | --- |
| Starchy vegetables |  |
| White potatoes | Baked, boiled, mashed, scalloped, and fried potatoes; potato chips; and mixtures having potatoes as a main ingredient, such as potato salad, stuffed baked potatoes, and potato soup, etc. |
| Other starchy vegetables | Immature peas, lima beans, corn, breadfruit, burdock, cassava, dasheen, green bananas, hominy, jicama, lotus root, parsnips, plantains, salsify, tannier, tapioca, and taro, etc. |
| Non-starchy vegetables |  |
| Dark-green vegetables | Arugula, basil, beet greens, bitter melon leaves, broccoli, Chinese cabbage (pak-choi), chrysanthemum garland, chard, cilantro, collards, cress, dandelion greens, kale, mustard cabbage, mustard greens, parsley, poke greens, spinach, turnip greens, and watercress, etc. |
| Red and orange vegetables | Calabaza (Spanish pumpkin), carrots, red chili peppers, red or orange bell peppers, pimento (pimiento), pumpkin, squash (most winter types), sweet potatoes, and tomatoes, etc. |
| Other non-starchy vegetables | Artichoke, asparagus, avocado, bamboo shoots, beans (green, string), bean sprouts, beets, bitter melon (bitter gourd, balsam pear), Brussels sprouts, cabbage (green, red, savoy), cauliflower, celeriac, celery, chives, cucumber, eggplant, fennel bulb, garlic, ginger root, kohlrabi, leeks, okra, olives, onions, snow peas, etc. |

Abbreviation: FPED, Food Patterns Equivalents Database.

**Table S2.** Characteristics of study participants by MetS status, weighted(n=24646) ^a^.

| **Characteristic** | **Overall population**  **(n=24646)** | **Non-MetS**  **(n=15828)** | **MetS**  **(n=8818)** | ***P* value** |
| --- | --- | --- | --- | --- |
| Age, years | 45.84±0.23 | 42.86±0.26 | 51.99±0.27 | < 0.001 |
| Gender, n (%) |  |  |  | < 0.001 |
| Female | 11725(47.57) | 7616(49.93) | 4109(45.93) |  |
| Male | 12921(52.43) | 8212(50.07) | 4709(54.07) |  |
| Race, n (%) |  |  |  | < 0.001 |
| Mexican American | 3814(15.48) | 2260(6.74) | 1554(8.05) |  |
| Non-Hispanic Black | 4562(18.51) | 3110(9.64) | 1452(7.94) |  |
| Non-Hispanic White | 12448(50.51) | 7944(72.67) | 4504(73.67) |  |
| Other races | 3822(15.51) | 2514(10.95) | 1308(10.34) |  |
| Education, n (%) |  |  |  | < 0.001 |
| Less than 9th grade | 1915(7.77) | 1041(3.02) | 874(4.58) |  |
| 9th-11th grade | 2967(12.04) | 1751(7.90) | 1216(10.34) |  |
| High school | 5641(22.89) | 3383(20.62) | 2258(27.11) |  |
| Some college | 7638(30.99) | 4931(32.25) | 2707(33.28) |  |
| College or above | 6485(26.31) | 4722(36.20) | 1763(24.68) |  |
| PIR, n (%) |  |  |  | < 0.001 |
| <1.30 | 6452(26.18) | 3956(17.82) | 2496(19.02) |  |
| 1.30-3.49 | 9221(37.41) | 5791(33.07) | 3430(36.21) |  |
| ≥3.50 | 8973(36.41) | 6081(49.11) | 2892(44.77) |  |
| Marital status, n (%) |  |  |  | < 0.001 |
| Unmarried | 13600(55.18) | 8312(55.10) | 5288(62.74) |  |
| Married | 13600(55.18) | 8312(55.10) | 5288(62.74) |  |
| Energy, kcal/d | 2151.27±7.77 | 2169.56± 9.35 | 2113.55±13.52 | < 0.001 |
| Fruits, g/day | 541.99±9.49 | 562.53±11.31 | 499.62±11.31 | < 0.001 |
| Carbohydrate, g/day | 257.53±1.06 | 252.92±1.79 | 259.77±1.42 | 0.005 |
| Fat, g/day | 82.50±0.39 | 82.61±0.44 | 82.28±0.68 | 0.664 |
| Whole grains, g/day | 191.84±3.85 | 196.12±4.59 | 183.03±4.86 | 0.023 |
| Refined grains, g/day | 799.03±7.08 | 793.64± 7.51 | 810.14±11.10 | 0.148 |
| Meat, g/day | 213.50±3.67 | 203.85±3.97 | 233.40±5.83 | < 0.001 |
| Nuts, g/day | 108.31±3.10 | 115.34±3.65 | 93.82±3.83 | < 0.001 |
| Coffee, g/day | 324.52±5.78 | 319.46±6.82 | 334.95±7.40 | 0.070 |
| Serum creatinine, umol/L | 78.06±0.23 | 76.77±0.24 | 80.70±0.42 | < 0.001 |
| Uric acid, μmol/L | 322.64±0.94 | 309.04±0.97 | 350.70±1.42 | < 0.001 |
| ALT, U/L | 25.91±0.18 | 24.10±0.19 | 29.65±0.42 | < 0.001 |
| AST, U/L | 25.41±0.13 | 24.86±0.16 | 26.54±0.24 | < 0.001 |
| Smoke, n (%) | 5014(20.34) | 3327(20.66) | 1687(18.93) | 0.041 |
| Physical activity (MET-  minute/week), n (%) |  |  |  | < 0.001 |
| <600 | 8746(35.49) | 5541(33.75) | 3205(35.40) |  |
| 600-1200 | 3694(14.99) | 2259(13.78) | 1435(16.15) |  |
| ≥1200 | 12206(49.53) | 8028(52.47) | 4178(48.45) |  |
| Alcohol status, n (%) |  |  |  | < 0.001 |
| Never | 6707(27.21) | 3702(19.06) | 3005(28.79) |  |
| Mild | 8890(36.07) | 5832(38.00) | 3058(37.19) |  |
| Moderate | 4056(16.46) | 2869(19.92) | 1187(15.09) |  |
| Heavy | 4993(20.26) | 3425(23.02) | 1568(18.94) |  |

Continuous variables were shown as mean±SE, categorical variables were shown as frequency(percentage)

^a^All estimates accounted for complex survey designs, and all percentages were weighted.

Abbreviations: MetS, metabolic syndrome; PIR, poverty income ratio; ALT, alanine aminotransferase; AST, aspartate aminotransferase; MET, metabolic equivalent; SE, standard error.
